# 2-Methyl­aspartic acid monohydrate

**DOI:** 10.1107/S1600536813032170

**Published:** 2013-11-30

**Authors:** Greg Brewer, Aaron S. Burton, Jason P. Dworkin, Ray J. Butcher

**Affiliations:** a Department of Chemistry, Catholic University of America, Washington, DC 20064, USA; b NASA Goddard Space Flight Center, Greenbelt, MD 20771, USA; c Solar System Exploration Division, NASA Goddard Space Flight Center, Greenbelt, MD 20771, USA; d Department of Chemistry, Howard University, 525 College Street NW, Washington, DC 20059, USA

## Abstract

The title compound, C_5_H_9_NO_4_·H_2_O, is an isomer of the α-amino acid glutamic acid that crystallizes from water in its zwitterionic form as a monohydrate. It is not one of the 20 proteinogenic α-amino acids that are used in living systems and differs from the natural amino acids in that it has an α-methyl group rather than an α-H atom. In the crystal, an O—H⋯O hydrogen bond is present between the acid and water mol­ecules while extensive N—H⋯O and O—H⋯O hydrogen bonds link the components into a three-dimensional array.

## Related literature

For the eighty amino acids that have been detected in meteorites or comets, see: Pizzarello *et al.* (2006 ▶); Glavin & Dworkin, (2009 ▶); Burton *et al.* (2012 ▶). For the role that crystallization plays in chiral separation, see: Blackmond & Klussmann (2007 ▶); Blackmond *et al.* (2008 ▶). For the role of the H atom on the α-C atom in enhancing the rate of racemization, see: Yamada *et al.* (1983 ▶). For the mechanism of racemization of amino acids lacking an α-H atom, see: Pizzarello & Groy (2011 ▶). For the role that crystallization can play in the enrichment of l isovaline and its structure, see: Glavin & Dworkin (2009 ▶); Butcher *et al.* (2013 ▶). For normal bond lengths and angles, see: Orpen (1993 ▶). For the number of α-methyl amino acids that have been observed with l-enanti­omeric excesses up to 20% that are not believed to be the result of contamination, see: Pizzarello & Cronin (2000 ▶); Glavin & Dworkin (2009 ▶); Glavin *et al.* (2011 ▶, 2012 ▶); Burton *et al.* (2013 ▶).
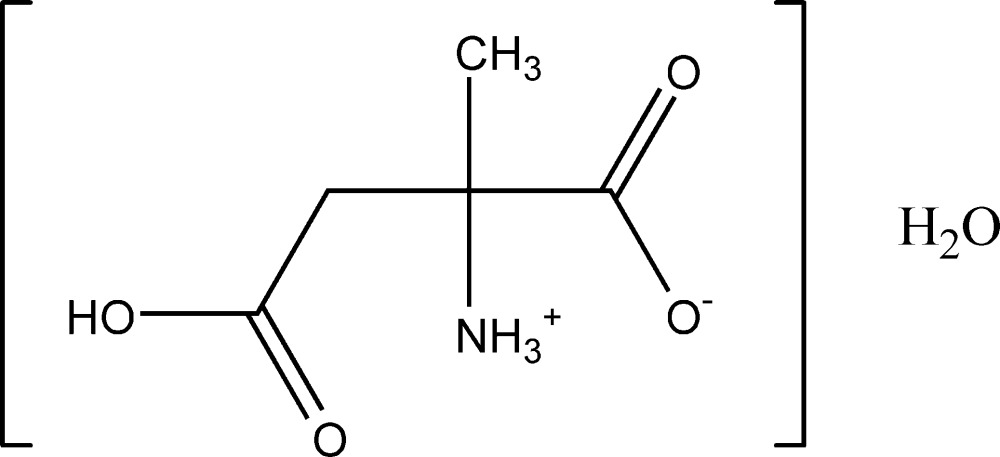


## Experimental

### 

#### Crystal data

C_5_H_9_NO_4_·H_2_O
*M* *_r_* = 165.15
Monoclinic, 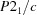

*a* = 9.9690 (6) Å
*b* = 12.8677 (6) Å
*c* = 5.8409 (3) Å
β = 106.491 (6)°
*V* = 718.44 (7) Å^3^
*Z* = 4
Cu *K*α radiation
μ = 1.20 mm^−1^
*T* = 123 K
0.49 × 0.12 × 0.04 mm

#### Data collection

Agilent Xcalibur Ruby Gemini diffractometer
Absorption correction: multi-scan (*CrysAlis PRO*; Agilent, 2012 ▶) *T*_min_ = 0.682, *T*_max_ = 1.000
5544 measured reflections
1498 independent reflections
1436 reflections with *I* > 2σ(*I*)
*R*_int_ = 0.038

#### Refinement

*R*[*F* ^2^ > 2σ(*F* ^2^)] = 0.090
*wR*(*F* ^2^) = 0.279
*S* = 1.20
1498 reflections
123 parameters
H atoms treated by a mixture of independent and constrained refinement
Δρ_max_ = 0.50 e Å^−3^
Δρ_min_ = −0.53 e Å^−3^

### 

Data collection: *CrysAlis PRO* (Agilent, 2012 ▶); cell refinement: *CrysAlis PRO*; data reduction: *CrysAlis PRO*; program(s) used to solve structure: *SHELXS97* (Sheldrick, 2008 ▶); program(s) used to refine structure: *SHELXL97* (Sheldrick, 2008 ▶); molecular graphics: *SHELXTL* (Sheldrick, 2008 ▶); software used to prepare material for publication: *SHELXTL*.

## Supplementary Material

Crystal structure: contains datablock(s) I, New_Global_Publ_Block. DOI: 10.1107/S1600536813032170/hg5362sup1.cif

Structure factors: contains datablock(s) I. DOI: 10.1107/S1600536813032170/hg5362Isup2.hkl

Supplementary material file. DOI: 10.1107/S1600536813032170/hg5362Isup3.cml

Additional supplementary materials:  crystallographic information; 3D view; checkCIF report

## Figures and Tables

**Table 1:** Hydrogen-bond geometry (Å, °)

*D*—H⋯*A*	*D*—H	H⋯*A*	*D*⋯*A*	*D*—H⋯*A*
O4—H4*O*⋯O1*W*	0.84 (8)	1.78 (8)	2.607 (4)	165 (7)
O1*W*—H1*W*1⋯O2^i^	0.89 (6)	1.83 (6)	2.705 (4)	168 (6)
O1*W*—H1*W*2⋯O3^ii^	0.82 (6)	2.09 (6)	2.909 (4)	174 (5)
N1—H1*A*⋯O1^iii^	0.91 (6)	1.93 (6)	2.807 (5)	164 (5)
N1—H1*B*⋯O2^iv^	0.90 (7)	1.94 (7)	2.832 (4)	172 (5)
N1—H1*C*⋯O3^v^	0.88 (6)	2.18 (6)	2.951 (4)	146 (5)
N1—H1*C*⋯O3	0.88 (6)	2.50 (6)	3.033 (4)	119 (5)

Symmetry codes: (i) 
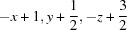
; (ii) 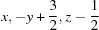; (iii) 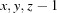; (iv) 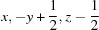; (v) 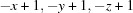.

**Fig. 1.**
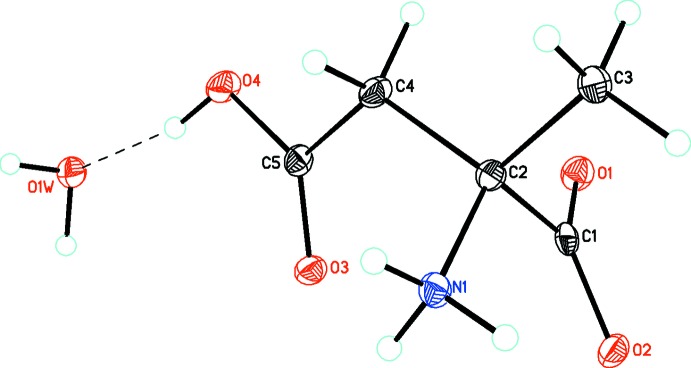
Diagram of the title compound showing atom labeling. Atomic displacement parameters are at the 30% probability level. Hydrogen bonds are shown as dashed lines.

**Fig. 2.**
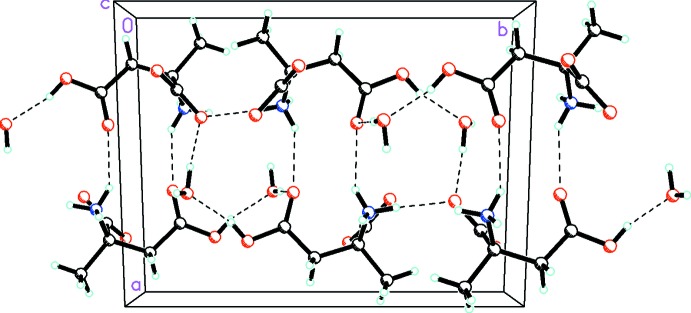
Packing diagram of the title compound viewed along the c axis showing the extensive N—H···O and O—H···O hydrogen bonds as dashed lines.

## supplementary crystallographic information

### 1. Comment

The α-amino acids are essential for life as they are the building blocks of all proteins and enzymes. Nature uses almost exclusively the *L* form of the nineteen common chiral amino acids. However, there are over eighty amino acids that have been identified in meteorites (Pizzarello *et al.*, 2006; Burton *et al.*, 2012). One of these extraterrestrial non-proteinogenic amino acids is 2-methylaspartic acid. The majority of meteoritic amino acids show little or no enrichment of one enantiomer over the other. However, a number of alpha methyl amino acids have been observed with *L*-enantiomeric excesses up to 20% that are not believed to be the result of contamination (Pizzarello & Cronin, 2000; Glavin & Dworkin, 2009; Glavin *et al.*, 2011; Glavin *et al.*, 2012; Burton *et al.*, 2013). An intriguing question is the process that leads to the separation and enrichment of the *L* enantiomer over the D. There are several possible explanations for this including the role that crystallization plays (Blackmond *et al.*, 2007; Glavin *et al.*, 2012). Only two of the twenty amino acids used biologically crystallize in a chiral space group from a racemic solution, which allows for spontaneous separation of enantiomers, at the level of the crystal (Blackmond *et al.*, (2008).

Racemic 2-methylaspartic crystallizes from water in an achiral space group acid, forming a racemic compound, in which there are equal numbers of D and *L* enantiomers in the unit cell. Thus, crystallization under these conditions would not provide a mechanism for separation of enantiomers at the level of the crystal. Another important aspect in the prebiotic chemistry of the amino acids is the role of racemization. All of the nineteen naturally occurring chiral amino acids have a hydrogen atom on the alpha carbon atom, which enhances the rate of racemization (Yamada *et al.*, 1983). However, little is known about the mechanism of racemization of amino acids lacking an alpha hydrogen atom (Pizzarello *et al.*, 2011). We recently reported the structure of another non-proteinogenic amino acid, isovaline, which crystallized as a racemic conglomerate from water in contrast to the present example which crystallizes in a centro-symmetric space group and is thus a racemate as indicated above (Butcher *et al.*, 2013). Resolved 2-methylaspartic acid and the structure given here can be used as a starting point in mechanistic studies of racemization mechanisms of amino acids lacking an alpha hydrogen atom.

In the structure of the title compound the amino acid is in the usual zwitterionic form involving the α carboxylate group and all the the bond lengths and angles are in the normal range for such compounds (Orpen, 1993). There is extensive N—H···O and O—H···O hydrogen bonding linking the zwitterions into a 3-D array.

### 2. Experimental

2-Methylaspartic acid was purchased from Nagase and Co. Ltd. Crystals of the title compound were grown from slow evaporation of a racemic solution of the amino acid in water.

### 3. Refinement

H atoms were placed in geometrically idealized positions and constrained to ride on their parent atoms with a C—H distances of 0.98 and 0.99 Å. The protons on the N and O were refined isotropically with the O—H distances for the water H's constrained to be 0.82 Å and the H—O—H angle close to 104.5°.

### Crystal data

**Table:**

C_5_H_9_NO_4_·H_2_O	*F*(000) = 352
*M**_r_* = 165.15	*D*_x_ = 1.527 Mg m^−^^3^
Monoclinic, *P*2_1_/*c*	Cu *K*α radiation, λ = 1.54178 Å
Hall symbol: -P 2ybc	Cell parameters from 3596 reflections
*a* = 9.9690 (6) Å	θ = 3.4–77.1°
*b* = 12.8677 (6) Å	µ = 1.20 mm^−^^1^
*c* = 5.8409 (3) Å	*T* = 123 K
β = 106.491 (6)°	Plate, colourless
*V* = 718.44 (7) Å^3^	0.49 × 0.12 × 0.04 mm
*Z* = 4	

### Data collection

**Table:**

Agilent Xcalibur Ruby Gemini diffractometer	1498 independent reflections
Radiation source: Enhance (Cu) X-ray Source	1436 reflections with *I* > 2σ(*I*)
Graphite monochromator	*R*_int_ = 0.038
Detector resolution: 10.5081 pixels mm^-1^	θ_max_ = 77.3°, θ_min_ = 3.4°
ω scans	*h* = −12→12
Absorption correction: multi-scan (*CrysAlis PRO*; Agilent, 2012)	*k* = −15→16
*T*_min_ = 0.682, *T*_max_ = 1.000	*l* = −7→5
5544 measured reflections	

### Refinement

**Table:**

Refinement on *F*^2^	Primary atom site location: structure-invariant direct methods
Least-squares matrix: full	Secondary atom site location: difference Fourier map
*R*[*F*^2^ > 2σ(*F*^2^)] = 0.090	Hydrogen site location: inferred from neighbouring sites
*wR*(*F*^2^) = 0.279	H atoms treated by a mixture of independent and constrained refinement
*S* = 1.20	*w* = 1/[σ^2^(*F*_o_^2^) + (0.171*P*)^2^ + 1.5626*P*] where *P* = (*F*_o_^2^ + 2*F*_c_^2^)/3
1498 reflections	(Δ/σ)_max_ < 0.001
123 parameters	Δρ_max_ = 0.50 e Å^−^^3^
0 restraints	Δρ_min_ = −0.53 e Å^−^^3^

### Special details

**Table:**

Geometry. All e.s.d.'s (except the e.s.d. in the dihedral angle between two l.s. planes) are estimated using the full covariance matrix. The cell e.s.d.'s are taken into account individually in the estimation of e.s.d.'s in distances, angles and torsion angles; correlations between e.s.d.'s in cell parameters are only used when they are defined by crystal symmetry. An approximate (isotropic) treatment of cell e.s.d.'s is used for estimating e.s.d.'s involving l.s. planes.
Refinement. Refinement of *F*^2^ against ALL reflections. The weighted *R*-factor *wR* and goodness of fit *S* are based on *F*^2^, conventional *R*-factors *R* are based on *F*, with *F* set to zero for negative *F*^2^. The threshold expression of *F*^2^ > σ(*F*^2^) is used only for calculating *R*-factors(gt) *etc*. and is not relevant to the choice of reflections for refinement. *R*-factors based on *F*^2^ are statistically about twice as large as those based on *F*, and *R*- factors based on ALL data will be even larger.

### Fractional atomic coordinates and isotropic or equivalent isotropic displacement parameters (Å^2^)

**Table:**

	*x*	*y*	*z*	*U*_iso_*/*U*_eq_	
O1	0.1948 (3)	0.4358 (2)	0.7672 (5)	0.0230 (7)	
O2	0.3545 (3)	0.3270 (2)	0.6994 (5)	0.0214 (6)	
O3	0.3796 (3)	0.5820 (2)	0.5612 (5)	0.0212 (6)	
O4	0.2105 (3)	0.6995 (2)	0.4131 (6)	0.0236 (7)	
H4O	0.276 (7)	0.743 (6)	0.457 (13)	0.042 (17)*	
O1W	0.3772 (3)	0.8584 (2)	0.5403 (6)	0.0246 (7)	
H1W1	0.469 (7)	0.851 (5)	0.608 (12)	0.033 (15)*	
H1W2	0.380 (6)	0.879 (4)	0.409 (11)	0.018 (12)*	
N1	0.3151 (3)	0.3888 (3)	0.2516 (6)	0.0183 (7)	
H1A	0.294 (6)	0.405 (5)	0.095 (11)	0.027*	
H1B	0.332 (6)	0.320 (5)	0.249 (10)	0.027*	
H1C	0.393 (6)	0.422 (5)	0.325 (11)	0.027*	
C1	0.2565 (4)	0.3913 (3)	0.6364 (7)	0.0182 (8)	
C2	0.2010 (4)	0.4134 (3)	0.3646 (7)	0.0174 (8)	
C3	0.0787 (4)	0.3399 (3)	0.2591 (7)	0.0198 (8)	
H3A	0.0458	0.3495	0.0857	0.030*	
H3B	0.0023	0.3552	0.3289	0.030*	
H3C	0.1095	0.2679	0.2950	0.030*	
C4	0.1559 (4)	0.5261 (3)	0.3097 (7)	0.0188 (8)	
H4A	0.1373	0.5379	0.1360	0.023*	
H4B	0.0671	0.5374	0.3502	0.023*	
C5	0.2607 (4)	0.6045 (3)	0.4406 (7)	0.0183 (8)	

### Atomic displacement parameters (Å^2^)

**Table:**

	*U*^11^	*U*^22^	*U*^33^	*U*^12^	*U*^13^	*U*^23^
O1	0.0262 (14)	0.0207 (14)	0.0234 (14)	0.0023 (11)	0.0091 (11)	0.0003 (11)
O2	0.0223 (13)	0.0144 (13)	0.0254 (13)	0.0015 (10)	0.0033 (11)	0.0024 (10)
O3	0.0178 (12)	0.0163 (12)	0.0275 (14)	0.0010 (10)	0.0032 (11)	0.0008 (11)
O4	0.0228 (13)	0.0124 (12)	0.0333 (15)	0.0024 (11)	0.0041 (12)	−0.0012 (11)
O1W	0.0222 (14)	0.0216 (14)	0.0288 (16)	−0.0019 (11)	0.0050 (12)	0.0042 (11)
N1	0.0189 (16)	0.0142 (15)	0.0225 (16)	−0.0011 (12)	0.0070 (12)	−0.0011 (12)
C1	0.0180 (17)	0.0103 (16)	0.0262 (19)	−0.0053 (12)	0.0063 (15)	−0.0004 (13)
C2	0.0167 (17)	0.0118 (15)	0.0242 (18)	0.0009 (13)	0.0065 (14)	−0.0002 (13)
C3	0.0200 (17)	0.0149 (16)	0.0232 (18)	−0.0032 (14)	0.0038 (14)	−0.0029 (14)
C4	0.0193 (17)	0.0141 (17)	0.0215 (16)	0.0034 (13)	0.0034 (14)	0.0005 (14)
C5	0.0212 (17)	0.0106 (16)	0.0244 (18)	0.0014 (13)	0.0087 (14)	−0.0005 (13)

### Geometric parameters (Å, º)

**Table:**

O1—C1	1.247 (5)	N1—H1C	0.88 (6)
O2—C1	1.253 (5)	C1—C2	1.552 (5)
O3—C5	1.229 (5)	C2—C4	1.525 (5)
O4—C5	1.313 (4)	C2—C3	1.528 (5)
O4—H4O	0.84 (8)	C3—H3A	0.9800
O1W—H1W1	0.89 (6)	C3—H3B	0.9800
O1W—H1W2	0.82 (6)	C3—H3C	0.9800
N1—C2	1.502 (5)	C4—C5	1.497 (5)
N1—H1A	0.91 (6)	C4—H4A	0.9900
N1—H1B	0.90 (7)	C4—H4B	0.9900
			
C5—O4—H4O	110 (5)	C3—C2—C1	108.1 (3)
H1W1—O1W—H1W2	99 (6)	C2—C3—H3A	109.5
C2—N1—H1A	114 (4)	C2—C3—H3B	109.5
C2—N1—H1B	112 (4)	H3A—C3—H3B	109.5
H1A—N1—H1B	102 (5)	C2—C3—H3C	109.5
C2—N1—H1C	111 (4)	H3A—C3—H3C	109.5
H1A—N1—H1C	108 (5)	H3B—C3—H3C	109.5
H1B—N1—H1C	110 (5)	C5—C4—C2	114.3 (3)
O1—C1—O2	127.0 (4)	C5—C4—H4A	108.7
O1—C1—C2	116.6 (3)	C2—C4—H4A	108.7
O2—C1—C2	116.3 (3)	C5—C4—H4B	108.7
N1—C2—C4	108.8 (3)	C2—C4—H4B	108.7
N1—C2—C3	108.0 (3)	H4A—C4—H4B	107.6
C4—C2—C3	110.5 (3)	O3—C5—O4	124.2 (3)
N1—C2—C1	108.5 (3)	O3—C5—C4	123.6 (3)
C4—C2—C1	112.8 (3)	O4—C5—C4	112.2 (3)
			
O1—C1—C2—N1	158.8 (3)	N1—C2—C4—C5	−71.6 (4)
O2—C1—C2—N1	−24.4 (4)	C3—C2—C4—C5	170.0 (3)
O1—C1—C2—C4	38.2 (4)	C1—C2—C4—C5	48.8 (4)
O2—C1—C2—C4	−145.0 (3)	C2—C4—C5—O3	8.0 (6)
O1—C1—C2—C3	−84.3 (4)	C2—C4—C5—O4	−171.5 (3)
O2—C1—C2—C3	92.6 (4)		

### Hydrogen-bond geometry (Å, º)

**Table:**

*D*—H···*A*	*D*—H	H···*A*	*D*···*A*	*D*—H···*A*
O4—H4*O*···O1*W*	0.84 (8)	1.78 (8)	2.607 (4)	165 (7)
O1*W*—H1*W*1···O2^i^	0.89 (6)	1.83 (6)	2.705 (4)	168 (6)
O1*W*—H1*W*2···O3^ii^	0.82 (6)	2.09 (6)	2.909 (4)	174 (5)
N1—H1*A*···O1^iii^	0.91 (6)	1.93 (6)	2.807 (5)	164 (5)
N1—H1*B*···O2^iv^	0.90 (7)	1.94 (7)	2.832 (4)	172 (5)
N1—H1*C*···O3^v^	0.88 (6)	2.18 (6)	2.951 (4)	146 (5)
N1—H1*C*···O3	0.88 (6)	2.50 (6)	3.033 (4)	119 (5)

Symmetry codes: (i) −*x*+1, *y*+1/2, −*z*+3/2; (ii) *x*, −*y*+3/2, *z*−1/2; (iii) *x*, *y*, *z*−1; (iv) *x*, −*y*+1/2, *z*−1/2; (v) −*x*+1, −*y*+1, −*z*+1.
